# Window entrapment trauma in cats: clinical, neurological and clinicopathological findings and outcome (70 cases)

**DOI:** 10.1177/1098612X241296416

**Published:** 2024-12-24

**Authors:** Fabiana Graciolli Tomazi, Veronika M Stein, Julia Hauer, Laureen M Peters, Frank Steffen, Dima Farra, Beatriz Vidondo, Arianna Maiolini

**Affiliations:** 1 Division of Clinical Neurology, Department of Clinical Veterinary Medicine, Vetsuisse Faculty, University of Bern, Bern, Switzerland; 2 Department of Neurology, Small Animal Clinic Hofheim, Hofheim am Taunus, Germany; 3 Clinical Diagnostic Laboratory, Department of Clinical Veterinary Medicine, Vetsuisse Faculty, University of Bern, Bern, Switzerland; 4 Division of Neurology, Department for Small Animals, Vetsuisse Faculty, University of Zurich, Zurich, Switzerland; 5 Veterinary Public Health Institute, Vetsuisse Faculty, University of Bern, Bern, Switzerland

**Keywords:** Ischaemic neuromyelomyopathy, window entrapment, Kippfenster syndrome, crush injury

## Abstract

**Objectives:**

Window entrapment in cats can lead to reduced blood flow to the spinal cord, muscles and nerves, resulting in ischaemic neuromyelomyopathy. The severity and duration of entrapment greatly influence clinical and neurological outcomes, as well as prognosis. The aim of the present retrospective multicentric study (2005–2022) was to describe clinical, neurological and selected clinicopathological findings, as well as the outcome of cats trapped in bottom-hung windows, presented to both first-opinion and referral-only clinics.

**Methods:**

The study included cats with detailed clinical and neurological evaluations at admission, along with at least one of the following biochemical parameters: creatine kinase (CK), aspartate transaminase (AST), alanine aminotransferase (ALT) activities, urea and/or creatinine. Clinical and neurological parameters evaluated in the study included rectal temperature, femoral pulse, gait, urinary bladder function, tail function and survival to discharge. Odds ratios (ORs) were calculated for survival and each clinical, neurological and biochemical variable.

**Results:**

Of the 70 cats that met the inclusion criteria, only seven (10%) died or were euthanased during hospitalisation. Nevertheless, with the available data, we found evidence of an association between clinical and neurological status and survival, with tail function being the strongest association. Cats lacking tail sensation, motor function and/or tonus were more likely to die than cats with normal tail function or only mild abnormalities (OR = 24). Similarly, cats with severe hypothermia or an absent femoral pulse were less likely to survive (OR = 12.75 and 7.5, respectively). In this sample (with a relatively low number of deaths), we did not find evidence of an association between CK, AST and ALT activity with survival. However, the only two cats with severe increases in creatinine died.

**Conclusions and relevance:**

Assessment of gait, urinary bladder function, femoral pulse, rectal temperature and particularly tail function is promising for predicting outcomes in cats with window entrapment trauma.

## Introduction

Window entrapment presents a notable risk of injury and even death for cats living in areas where bottom-hung windows are common, particularly in the Germanic countries, such as Germany, Switzerland and Austria. These windows, often referred to as ‘tilt’ or ‘hopper’ windows, have a vertical opening that tilts inward.^1^ The associated condition has been referred to as ‘Kippfenster-Syndrom’ in the German literature^2^ or as ‘bottom-hung window trauma’ in a previous study.^3^

The trauma occurs when cats become accidentally trapped, often around the thoracolumbar area, leading to varying degrees of organic hypoperfusion that can affect various structures, including the spinal cord, muscles, nerves and kidneys, depending on the severity of the trauma. Neurological abnormalities resulting from this condition can range from mild deficits to complete paralysis. Typically, both pelvic limbs are affected; however, deficits in a single limb or all four limbs are also possible, depending on whether the entrapment occurs in one limb or the cervical region, respectively. The neurological signs are most likely derived from a combination of vessel compression, leading to spinal cord and muscle ischaemia, and direct spinal nerve compression, resulting in neurapraxia or axonotmesis.^3^ The clinical presentation resembles that of feline aortic thromboembolism and traumatic ischaemic myelopathy, where the main vessel occlusion is due to a thrombus or blunt abdominal trauma, respectively.^4,5^

Another serious consequence of traumatic entrapment-induced ischaemia is reperfusion injury, leading to tissue damage and possibly cell death, commonly resulting in sudden respiratory distress.^6–9^ Ischaemia-reperfusion injury can cause multiple organ dysfunction or failure.^10,11^

In human medicine, a condition frequently observed during catastrophic events such as earthquakes is termed ‘crush injury/syndrome’. This condition occurs when a limb or other body part is trapped under heavy objects, causing continuous pressure so intense that it surpasses capillary perfusion pressure, preventing blood flow and leading to tissue ischaemia. Upon removal of compression, muscle and neurological disturbances may occur, along with severe systemic manifestations, including rhabdomyolysis, systemic organ dysfunction and acute kidney injury.^12^

In human cases of traumatic rhabdomyolysis, creatine kinase (CK) levels serve as a screening biomarker, while acute kidney injury is diagnosed based on azotaemia and acid–base abnormalities.^13–15^ Animal studies inducing reperfusion injury, such as hindlimb crushing in dogs, have demonstrated significant impairment of renal function.^16^ In addition, research in dogs, especially concerning renal transplantation, highlights oxidative stress as a critical factor in renal injuries.^11,17^ In rabbits, hindlimb ischaemia induced by a tourniquet leads to elevated blood urea nitrogen levels after reperfusion, with cases involving both extremities sometimes resulting in mortality due to hypovolaemic shock.^18^

At veterinary clinics and smaller practices, biochemical biomarkers, such as creatine kinase (CK), aspartate transaminase (AST), alanine aminotransferase (ALT), urea and creatinine, are routinely included in blood profiles to assess organ function in cats. These biomarkers serve as both direct and indirect indicators of muscle damage and renal function.^19–22^ Given the potential hypoperfusion of muscles and kidneys during entrapment, these markers are crucial for evaluating injury severity.^23^ Entrapment incidents can lead to spinal cord injury, resulting in tail dysfunction and often neurogenic bladder dysfunction, which may progress to bacterial cystitis and, in severe cases, renal dysfunction.^24^ Hypothermia, which can ensue from hypovolemic shock, can profoundly affect nervous system function and various organ systems, particularly the cardiovascular system, potentially reducing renal blood flow.^23^

Publications on window entrapment in cats are scarce, likely owing to its limited geographical occurrence.^2^ Only one retrospective study is available in English.^3^ Two previous studies have described the clinical, neurological, clinicopathological, radiological findings, follow-up and outcome of entrapped cats, investigating the influence of neurological status on the mortality rate of admitted paraplegic/paraplegic cats, with reported death rates in the range of 25–35%. Both studies were based on cases of cats presented at university referral centres; however, there was no description of the correlation between clinicopathological, clinical and neurological findings and outcome of cases presented to both referral and first-opinion veterinary centres.^2,3^

The aim of the present study was to describe the clinical, neurological and clinicopathological findings and outcomes of cats with window entrapment trauma presented to either referral-only or first-opinion centres. In addition, we investigated associations between blood biomarkers and clinical and neurological findings and survival outcome.

## Materials and methods

### Selection criteria

Digital medical records were searched at the Small Animal Clinics of the Vetsuisse Faculties, University of Bern and Zurich (Switzerland) and Small Animal Clinic of Hofheim (Germany) between July 2005 and December 2022 for cats with a history of being found entrapped in a bottom-hung window, undergoing neurological and clinical examination and having serum chemistry performed for at least one of the following parameters: CK, AST, ALT, urea and creatinine. Search terms used to identify relevant cases included ‘ischaemic neuromyelopathy’, ‘ischaemic myelopathy’, ‘ischaemic neuromyopathy’, ‘ischaemic neuromyelomyopathy’, ‘bottom-hung window’ and ‘Kippfenster syndrome’. Cases lacking recorded neurological or clinical data or without blood analysis for at least one of the specified parameters were excluded from the study.

### Clinical assessment and classification

The following clinical parameters were assessed on admission: rectal temperature (in °C), heart rate (HR), respiratory rate (RR) and bilateral femoral pulse. The estimated duration of entrapment was recorded, when available. Cats with comorbidities that could potentially alter clinical assessment (eg, congestive heart failure, acute or chronic renal failure, history of long-term glucocorticoid use, blood pressure medication, etc) were not included. Rectal body temperature and femoral pulse were key clinical markers of interest in the study; therefore, cats were classified in groups according to the severity of abnormalities.

Cats were classified as being normothermic (⩾37.8°C ⩽39.5°C), mildly hypothermic (⩾36.7°C ⩽37.7°C), moderately hypothermic (⩾35.5°C <36.7°C) or severely hypothermic (<35.5°C).^25,26^

Cats were classified as having a normal, reduced (detectable but weak) or absent pulse. In cases of asymmetrical findings, cats were classified according to the worst side.

### Neurological assessment and classification

The neurological assessment was based on the information retrieved from the neurological examination concerning the gait, urinary bladder function and tail function.

Gait was scored as normal, ambulatory paretic, non-ambulatory paretic, plegic with deep pain perception (DPP) or plegic without DPP.^27^

In terms of urinary bladder function, cats were categorised as either voluntary micturition (able to urinate without assistance) or involuntary micturition (unable to urinate without assistance).^28^

Tail function was categorised as normal, reduced or absent based on reported abnormalities in motor function, sensation and/or tone.

### Selected clinicopathologic data (CK, AST, ALT, urea and creatinine)

The serum chemistry values were extracted from the medical records for the following analytes: CK, AST, ALT, urea and creatinine. Biochemical analyses were performed on lithium-heparinised plasma using commercial biochemistry analysers at the following laboratories: the Central Diagnostic Laboratory of the Vetsuisse Faculty, University of Bern utilised 911 Chemistry Analyser (Roche Hitachi) before 8 October 2012 and cobas c501 (Roche Diagnostics) after 8 October 2012; the Central Diagnostic Laboratory of the Vetsuisse Faculty, University of Zurich also utilised the cobas c501 biochemistry analyser (Roche/Hitachi); and the laboratory in Hofheim used Vet Test 8008 (IDEXX) before 2010, DRI-Chem FDC NX 700 (Fuji) after 2010 and Cobas Integra 400 plus (Roche) since 2018. To enhance comparability across different laboratories, values were categorised according to their degree of increase relative to the upper reference limit (URL) specific to each laboratory for CK, AST, ALT, urea and creatinine.^20,29,30^

CK activity was categorised based on the degree of increase relative to its URL: normal (⩽URL), mildly (>1 to <5-fold URL), moderately (⩾5 to <30-fold URL) or severely increased (⩾30-fold URL).^20,29^

AST and ALT activities were categorised as follows: normal (⩽URL), mildly (<5-fold URL), moderately (⩾5 to <10-fold URL) or severely increased (>10-fold URL).^29^

Urea values were classified as follows: normal (below URL) or increased, with increases further categorised as mildly (>1 to <2-fold URL), moderately (⩾2 to ⩽3-fold URL) or severely increased (>3-fold URL).^30^

Creatinine levels were categorised according to the modified grading criteria of the International Renal Interest Society (IRIS) for acute kidney injury (AKI) as follows: normal (⩽140 μmol/l ), mild (>140 to ⩽220 μmol/l), moderate (>220 ⩽440 μmol/l) or severe (>440 μmol/l) increase.^31^

### Outcomes

Short-term outcomes were evaluated for all cats until the time of discharge. Cats were divided into two groups: survivors and non-survivors. Survivors referred to cats that were alive at the time of discharge, whereas non-survivors encompassed cats that were either euthanased or died during hospitalisation.

### Statistical analysis

**Figure 1:**
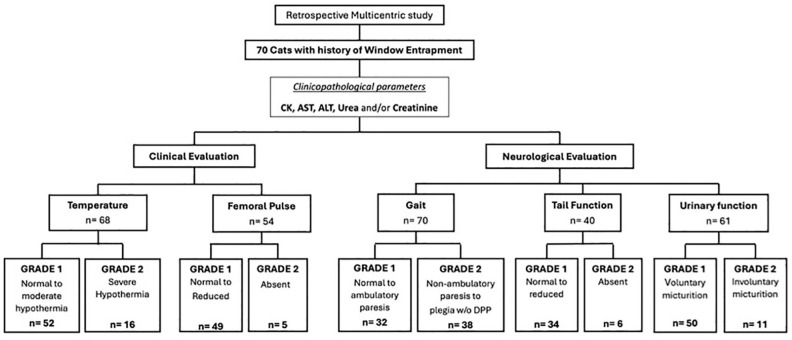
Schematic overview on the selection and clinical classification of cats included in the study. ALT = alanine aminotransferase; AST = aspartate transaminase; CK = creatine kinase; DPP = deep pain perception; w/o = without

**Figure 2:**
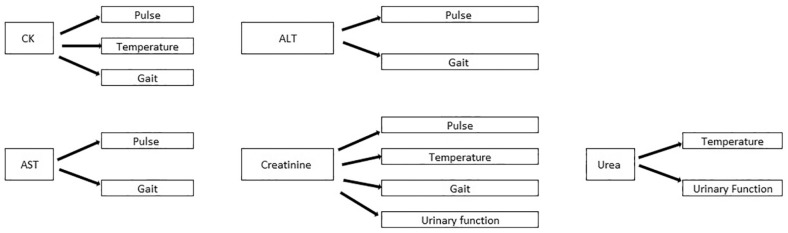
Schematic overview of the biologically meaningful associations (pairs of biochemical, neurological and clinical variables) investigated in this study. ALT = alanine aminotransferase; AST = aspartate transaminase; CK = creatine kinase

In order to facilitate a comparison between severity, the population was divided into two grades: grade 1, which included the milder cases; and grade 2, which included the more severe cases (Figure 1). All variables including the outcome (survivors vs non-survivors) were defined as binary variables (as described above). Statistical associations between biologically meaningful variables (biochemical, neurological and clinical) were investigated (Figure 2). The descriptive analysis included cross-tables. Odds ratios (ORs) were calculated for survival and each clinical, neurological and biochemical variable (whenever there were sufficient numbers of survivors and non-survivors). Mantel–Haenszel tests were used to test the two-sided null hypothesis of whether the odds ratios were different from 1. Statistical analyses were performed using NCSS version 2023.^32^

## Results

A total of 70 cats met the inclusion criteria and were further assessed and classified as shown in the flow chart (Figure 1).

### Signalment and clinical findings

The majority of cats were young cats with a median age of 2 years (range 4 months to 15 years), the most common breed represented was domestic shorthair (n = 61), followed by British Shorthair (n = 4), Persian (n = 2), Birman (n = 1), Siamese (n = 1) and Neva Masquerade (n = 1). A total of 20 (29%) entire females, 16 (23%) neutered females, 14 (20%) entire males, 17 (24%) neutered males and three (4%) cats with no recorded sex were included. Duration of entrapment (information available in 44/70 cats) varied from minutes to up to 12 h. An assessment of clinical parameters on admission revealed 68 cats had their temperature recorded and the majority (n = 47, 69%) were hypothermic (⩽37.7°C). Femoral pulse assessment was available in 54 cats, of which 27 (50%) were normal (Table 1).

**Table 1:** Results of the parameters of interest of the clinical and neurological examination, classification according to severity, outcome expressed as survival at discharge and ORs

		Number of cats	Severity degree	Survivors	Non-survivors	OR	*P* value	*P*1	*P*2
Clinical examination									
Rectal body temperature (68/70)	(⩾37.8°C ⩽39.5°C)	21 (31)	1	51/53 (96)	2/53 (4)	12.75	0.009	0.33	0.04
	Mildly hypothermic (⩾36.7°C ⩽37.7°C)	15 (22)							
	Moderately hypothermic (⩾35.5°C <36.7°C)	16 (23.5)							
	Severe hypothermic (<35.5°C)	16 (23.5)	2	10/15 (67)	5/15 (33)				
Femoral pulse (54/70)	Normal	27 (50)	1	45/48 (94)	3/48 (6)	7.5	0.03	0.33	0.06
	Reduced	22 (41)							
	Absent	5 (9)	2	4/6 (67)	2/6 (33)				
Neurological examination									
Gait (70/70)	Normal	5 (7)	1	32/32 (100)	0	72259	0.9	0.18	–
	Ambulatory paraparesis	26 (37)							
	Ambulatory tetraparesis	1 (1.5)							
	Non-ambulatory paraparesis	22 (31.5)	2	31/38 (81)	7/38 (18)				
	Paraplegia with deep pain perception	6 (9)							
	Paraplegia without deep pain perception	10 (14)							
Tail function (40/70)	Normal (motoric, sensation, tonus)	27 (67)	1	32/33 (97)	1/33 (3)	24	0.014	0.4	0.03
	Reduced (motoric, sensation, tonus)	7 (18)							
	Absent (motoric, sensation, tonus)	6 (15)	2	4/7 (57)	3/7 (43)				
Urinary function (61/70)	Voluntary micturition	50 (82)	1	42/42 (100)	0	105004	0.0031	0.2	–
	Involuntary micturition	11 (18)	2	8/10 (80)	2/10 (20)				

Data are n (%) unless otherwise indicated. *P* value significance level is set at 0.05; *P*1 represents the proportion of cats with grade 2 that survived, while *P*2 represents the proportion of cats with grade 1 that did not survive

OR = odds ratio

### Neurological findings

Gait was assessed in all cats, with the majority being paraparetic; 26 (37%) cats were ambulatory and 22 (31%) cats were non-ambulatory. Tail function (motor, sensation and/or tone) was assessed in 40 (57%) cats and was normal in 27 (67%) cats. Urinary bladder function was assessed in 61 (87%) cats, with 50 (82%) cats showing voluntary micturition (Table 1).

### Clinicopathological findings

CK activity was increased in all 22 (100%) cats with CK recorded, demonstrating a severe increase in the majority (17/22, 77%). AST activity was measured in 33 cats and severely increased in 16 (49%) cats. ALT activity was available in 42 cats, being mildly increased in 28 (67%).

Urea was measured in 66 cats and nearly half of them (31/66, 47%) showed increased urea concentration. Creatinine concentration was measured in 69 cats, showing increased levels in 25 (36%) cats (Table 2).

**Table 2:** Results of the clinicopathological parameters of interest, classification according to severity of increase and outcome expressed as survival at discharge

Parameter and classification		Number of cats	Severity degree	Survivors	Non-survivors
CK (22/70)	Normal (⩽URL)	0 (0)	1	4/5 (80)	1/5 (20)
	Mild (>1 <5-fold URL)	1 (5)			
	Moderate (⩾5 <30-fold URL)	4 (18)			
	Severe (⩾30-fold URL)	17 (77)	2	17/17 (100)	0
AST (33/70)	Normal (⩽URL)	2 (6)	1	16/17 (94)	1/17 (6)
	Mild (>1 <5-fold URL)	11 (33)			
	Moderate (⩾5 ⩽10-fold URL)	4 (12)			
	Severe (>10-fold URL)	16 (49)	2	15/16 (94)	1/16 (6)
ALT (42/70)	Normal (⩽URL)	10 (24)	1	37/41 (90)	4/41 (10)
	Mild (>1 <5-fold URL)	28 (67)			
	Moderate (⩾5 ⩽10-fold URL)	3 (7)			
	Severe (>10-fold URL)	1 (2)	2	1/1 (100)	0
Urea (66/70)	Normal (<URL)	35 (53)	1	57/62 (92)	5/62 (8)
	Mild (⩾1 ⩽2-fold URL)	25 (38)			
	Moderate (>2 ⩽3-fold URL)	2 (3)			
	Severe (>3-fold URL)	4 (6)	2	3/4 (75)	1/4 (25)
Creatinine (69/70)	Normal (⩽140 μmol/l)	44 (64)	1	62/67 (93)	5/67 (7)
	Mild (>140 ⩽220 μmol/l)	17 (24)			
	Moderate (>220 ⩽440 μmol/l)	6 (9)			
	Severe (>440 μmol/l)	2 (3)	2	0	2/2 (100)

Data are n (%)

ALT = alanine aminotransferase; AST = aspartate transaminase; CK = creatine kinase; URL = upper reference limit

### Outcomes

A total of 63 (90%) cats were survivors and were discharged from the clinic with either improved (61/63), stable (1/63) or mildly worsened (1/63) neurological status, while seven (10%) cats were non-survivors. Of the latter, the majority (five cats, 72%) died or were euthanased on the day of admission, whereas one (14%) cat died the following day and another (14%) was euthanased after 3 days. The causes of death/euthanasia included clinical deterioration (n = 2), lack of improvement, severe dyspnoea, cardiac arrest, coma and poor prognosis (one cat each).

With these available data, we found evidence of an association between clinical and neurological status and survival, with tail function being the strongest variable. Cats lacking tail sensation, motor function and/or tonus were 24 times more likely to die than cats with normal tail function or only mild abnormalities. Similarly, cats with severe hypothermia or an absent femoral pulse were 12.75 and 7.5 times less likely to survive, respectively.

The percentage of survivors based on body temperature at admission was 96% for temperature below 35.5°C (grade 1) and 67% for those with temperature of 35.5°C or higher (grade 2). When evaluating cats according to the presence/absence of pulse, the percentage of survivors with an absent pulse (grade 1) and a normal to reduce pulse (grade 2) was 94% and 67%, respectively.

Regarding gait, all ambulatory cats (grade 1) survived, whereas 18% of non-ambulatory to plegic cats (grade 2) died. Moreover, paraplegic cats without DPP had the highest percentage of deaths (67%).

Similarly, all cats showing voluntary micturition (grade 1) survived, while 80% of those without voluntary micturition (grade 2) survived. The percentage of survivors with normal or only mild tail abnormalities was 97% in contrast with only 57% for those cats with absent tail sensation, motor function and/or tonus (Table 1).

In this sample (with a relatively low number of deaths), we did not find evidence of association of CK, AST and ALT activity with survival; however, the only two cats with severe increases in creatinine died (Table 2).

### Association between clinicopathological and clinical/neurological findings

An overview of the distribution of biochemical parameter levels (CK, AST, ALT, urea and creatinine) across different grades of clinical variables (gait, temperature, pulse and urinary function) is provided (Table 3). Cats with severe hypothermia (grade 2) had severely increased creatinine concentrations, whereas only a minority had severe increases in CK activity. In this sample, no evidence of associations was found between the presence/absence of femoral pulse and clinicopathological findings. The majority of cats with severely increased CK and AST activities as well as severe azotaemia were non-ambulatory. The majority of cats with severely increased concentrations of urea had voluntary micturition, whereas the only cat with severely increased creatinine was unable to urinate without assistance (Table 3).

**Table 3:** Distribution of clinicopathological results based on clinical and neurological findings

Variables		CK		AST		ALT		Urea		Creatinine	
		Grade 1 (n = 5)	Grade 2 (n = 17)	Grade 1 (n = 17)	Grade 2 (n = 16)	Grade 1 (n = 41)	Grade 2 (n = 1)	Grade 1 (n = 62)	Grade 2 (n = 4)	Grade 1 (n = 67)	Grade 2 (n = 2)
Temperature	Grade 1 (n = 52)	4/5 (80)	12/17 (71)	NE	NE	NE	NE	47/60 (78)	2/4 (50)	52/65 (80)	0/2 (0)
	Grade 2 (n = 16)	1/5 (20)	5/17 (29)	NE	NE	NE	NE	13/60 (22)	2/4 (50)	13/65 (20)	2/2 (100)
Femoral pulse	Grade 1 (n = 49)	2/2 (100)	10/13 (77)	13/14 (93)	7/10 (70)	27/30 (90)	1/1 (100)	NE	NE	47/52 (90)	1/2 (50)
	Grade 2 (n = 5)	0/2 (0)	3/13 (23)	1/14 (7)	3/10 (30)	3/30 (10)	0/1 (0)	NE	NE	5/52 (10)	1/2 (50)
Gait	Grade 1 (n = 32)	2/5 (40)	6/17 (35)	8/17 (47)	4/16 (25)	17/41 (41)	1/1 (100)	NE	NE	31/67 (46)	0/2 (0)
	Grade 2 (n = 38)	3/5 (60)	11/17 (65)	9/17 (53)	12/16 (75)	24/41 (59)	0/0 (0)	NE	NE	36/67 (54)	2/2 (100)
Urinary function	Grade 1 (n = 50)	NE	NE	NE	NE	NE	NE	39/45 (87)	3/4 (75)	41/50 (82)	0/1 (0)
	Grade 2 (n = 11)	NE	NE	NE	NE	NE	NE	6/45 (13)	1/4 (25)	9/50 (18)	1/1 (100)

Data are n (%). Clinicopathological variables: grade 1 = normal to moderately increased values; grade 2 = severely increased values. Temperature: grade 1 = normal to moderate hypothermia; grade 2 = severe hypothermia. Femoral pulse: grade 1 = normal to reduced; grade 2 = absent. Gait: grade 1 = normal to ambulatory paresis; grade 2 = non-ambulatory paresis to plegia. Urinary function: grade 1 = presence of voluntary micturition; grade 2 = absence of voluntary micturition

ALT = alanine aminotransferase; AST = aspartate transaminase; CK = creatine kinase; NE = not evaluated

## Discussion

In this multicentric retrospective study, we focused on describing clinical, neurological and clinicopathological findings and their association with outcomes in a large cohort of cats with bottom-hung window entrapment trauma seen at both university referral centres and private primary and referral clinics.

We found a low proportion of deaths, at only 10%. Previous studies, based on cases referred exclusively to universities, reported mortality rates 2–3 times higher, in the range of 25–35%.^2,3^ The first study described clinicopathological findings in entrapped cats, and no associations with outcomes were tested.^2^ The second study analysed the influence of neurological and clinical parameters on survival excluding clinicopathological findings.^3^ Both studies were based on cases referred exclusively to universities.^2,3^ This percentage based solely on referrals to university clinics may not accurately represent the overall prognosis for this disease, as different practice settings, diagnostic and therapeutic resources, as well as owner compliance, can all influence outcomes. It is reasonable to assume that more severe cases are more likely to be referred to university hospitals, whereas less severe cases tend to remain in primary care centres, potentially resulting in a biased outcome. Indeed, our population included more cats that were still ambulatory compared with previous studies.^2,3^ When considering only cats lacking DPP, the previously reported mortality rate was 55%,^3^ which is comparable with the 67% observed in the present study.

Another possible reason for the lower mortality in our study is that we only included patients with available biochemical and neurological data, and those with a lower prognosis might have been euthanased before these data could be gathered. A further reason for the low overall percentage of deaths could be that only short-term outcomes were evaluated; therefore, it is possible that some cats that were discharged might have been euthanased later. However, the majority of cats showed an improved neurological status at discharge, and complete recovery has been previously documented to occur within a timeframe of 2–35 days.^2^ This makes euthanasia after initial recovery highly unlikely.

As a result of the limited number of euthanased cats in our sample (given our case definition), calculating ORs for each clinical, neurological and clinicopathological parameter was not always possible. Nevertheless, we found evidence of an association between clinical and neurological status and survival, with tail function being the strongest association. All cats maintaining ambulatory function or unassisted micturition survived. This association should be interpreted with caution, as the severity of certain clinical parameters may have influenced the decision for euthanasia, rather than predicting a worse outcome.

The blood parameters analysed in this study were selected based on their relevance to entrapment trauma, particularly ischaemic reperfusion injury and ischaemic neuromyopathy. Furthermore, basic biochemical parameters were selected that are readily available in private practice settings.

We found that CK activity was severely increased in the majority of the presented cats, whereas half of the cats had severely increased AST levels and ALT activity was predominantly within the normal limits or mildly increased. These findings are expected, as CK is the most specific enzyme for skeletal muscle damage, which peaks very early after the insult, followed by AST, which can also be found in hepatocytes and has a longer half-life. On the other hand, ALT, which is found predominantly in the liver, is expected to increase only mildly with severe skeletal muscle injury.

Among the 22 cats where CK activity was available, only one cat died, and this cat had only moderate increased CK activity. Furthermore, the severity of the CK increase was similar among ambulatory and non-ambulatory cats. This might be due to the very short half-life of CK, meaning that the peak of this enzyme activity may have been missed in cats where the blood sample was taken several hours after the documented entrapment incident. A correlation with the time and duration of the entrapment was not performed in this study, as in most cases, only an approximate estimate of the time of entrapment was available. Similarly, of the four cats that did not survive and for which ALT activities were available, all were grade 1. In the group of cats for which AST activities were available, two cats died, one in each grade.

Severe increases in creatinine and urea were not common, but the few cats classified as grade 2 died, making these parameters potential biomarkers for negative prognosis. This likely reflects a kidney injury sustained during the entrapment. Notably, the cases with more severe increases in creatinine and urea concentrations also showed more severe clinical and neurological signs.

Our multicentre study was of a retrospective nature, leading to variability in the clinical and neurological assessments performed by different clinicians, not all of them being neurology specialists. Limiting the neurological examination to assess only gait, urinary function and tail function potentially reduced variability in interpretation. In addition, the main groups were categorised in an easily assessable manner, such as distinguishing those that could walk without assistance. Biochemical analysers and types of parameters analysed differed among the cats, as well as the time between the trauma and blood sampling. This inevitably affected our results. Future studies should focus on both collecting long-term data, recruiting higher numbers of death cases and re-testing associations as a case-control study design. Comparative investigations, evaluating therapeutic interventions, exploring pathophysiological mechanisms, studying biochemical markers, fostering multicentric collaborations and implementing standardised assessment protocols are also necessary for a better understanding and management of this type of patients.

## Conclusions

The assessment of clinical parameters, such as gait, urinary bladder function, femoral pulse, rectal temperature and particularly tail function, seems promising for predicting outcome on patients with window entrapment syndrome.
